# Weekly Paclitaxel given concurrently with Durvalumab has a favorable safety profile in triple-negative metastatic breast cancer

**DOI:** 10.1038/s41598-021-98113-6

**Published:** 2021-09-27

**Authors:** Hazem Ghebeh, Adher Al-Sayed, Riham Eiada, Leilani Cabangon, Dahish Ajarim, Kausar Suleman, Asma Tulbah, Taher Al-Tweigeri

**Affiliations:** 1 Research Centre, King Faisal Specialist Hospital and Research Centre, Riyadh, Saudi Arabia; 2 College of Medicine, Al-Faisal University, Riyadh, Saudi Arabia; 3 Oncology Centre, King Faisal Specialist Hospital and Research Centre, Riyadh, Saudi Arabia; 4 Department of Radiology, King Faisal Specialist Hospital and Research Centre, Riyadh, Saudi Arabia; 5 Department of Pathology and Laboratory Medicine, King Faisal Specialist Hospital and Research Centre, Riyadh, Saudi Arabia

**Keywords:** Cancer, Cancer, Cancer, Immunology, Diseases, Medical research, Oncology

## Abstract

Therapeutic anti-PD-L1 antibodies are safe as a monotherapy, albeit with minimal efficacy in triple-negative breast cancer (TNBC). This trial aimed to test the safety and efficacy of Durvalumab and Paclitaxel in metastatic TNBC. In this open-label, one-arm trial, five cycles of weekly paclitaxel were delivered intravenously (IV) concurrent with Durvalumab that was given IV every 2 weeks. The combination was preceded by one cycle of paclitaxel alone, for immunological priming, followed by Durvalumab solo until disease progression or unacceptable toxicity. Between 2017 and 2019, 14 patients received at least one cycle of the combination therapy. The therapy was safe with no-dose limiting toxicity, except one case of skin lesions. Adverse events (AEs) were reported in 71% of patients, and there was no death due to the combination therapy. Regardless of grade, the most common AEs were headache and peripheral neuropathy, as each happened in four patients (29%), followed by fatigue and skin rash in three patients (21%) each. Grade 3/4 AEs were experienced by three patients (21%), with the most common being headache and anemia, which happened in two patients (14%). The confirmed objective response rate (ORR) was observed in five patients with a median duration of 10.0 months. Median Progression-free survival (PFS) and overall survival (OS) were 5 and 20.7 months, respectively. The combination of Durvalumab and Paclitaxel is safe, leaving room for additional agents. This is the first report on the combination of Durvalumab and Paclitaxel in the treatment of TNBC (NCT02628132).

## Introduction

Breast cancer is the most common cancer in women and second in terms of mortality^1^. There is evidence that breast cancer can be immunogenic, as evidenced by the infiltration of different types of immune cells^2^. Specifically, a subtype that is negative for estrogen and progesterone receptors and lacks Her2/neu overexpression, called triple-negative breast cancer (TNBC), is relatively more immunogenic^3^. However, immune-suppressive factors from tumor cells and their microenvironment can lead to tumor escape^4^.

Programmed cell death-1 (PD-1) and its ligand, Programmed cell death ligand-1 (PD-L1) axis is an important T-cell inhibitory checkpoint that acts at multiple sites in the body to control and regulate normal immune responses^5^. However, it is exploited by tumors to evade detection and elimination by the host immune system^6^. We have previously demonstrated the expression of PD-L1 in breast cancer and its correlation with well-known bad prognostic factors, including hormone receptor negativity status and high histological grade^7^. Other subsequent studies have shown PD-L1 expression to be enriched in TNBC^8^, while PD-L1 targeting as a monotherapy or in combination with other agents was feasible and promising^9,10^.

In patients with metastatic TNBC, anti-PD-L1 therapeutic agents had shown very limited efficacy in monotherapy settings as the reported objective response rate (ORR) was limited to 5–10% for unselected patients^11,12^, while ORR increased up to 21% in PD-L1 positive patients^13^. Recently, it has been shown that a combination of anti-PD-L1 targeting therapy (atezolizumab) with chemotherapy (Nab-paclitaxel) results in ORR up to 56%^10^.

Durvalumab, a human monoclonal antibody (MAb), functions as an immune checkpoint inhibitor by blocking the binding of PD-L1 to its receptors, PD-1 and CD80 (B7-1), in order to reactivate tumor-specific T-cell response and enhance antitumor activity. Durvalumab is approved by the US Food and Drug Administration (FDA) to treat several types of urothelial and lung cancers^14^. On the other hand, paclitaxel is one of the active chemotherapeutic agents that is commonly used to treat metastatic breast cancer^15^. However, the safety of paclitaxel, in combination with Durvalumab, has not been established yet.

We anticipated that the combination of Durvalumab with Paclitaxel would be safe and tolerable with minimal side events. We hypothesized that Durvalumab and Paclitaxel would synergistically control TNBC based on previous reports showing upregulation of PD-L1 by paclitaxel^16,17^ and the synergistic effect of chemotherapeutic agents and PD-L1 inhibition on promoting cancer cell apoptosis^18,19^. In addition, paclitaxel promotes the development of tumor-infiltrating lymphocytes (TIL)^20,21^, while the response to anti-PD-L1 therapy correlates with pre-existing tumor immunity like CD8+ infiltrating cells and TH1-type CD4+ activated lymphocytes^22^.

In this report, we have shown, for the first time, that indeed the combination of Paclitaxel and Durvalumab was safe with no dose-limiting toxicity. The activity was promising but inconclusive.

## Patients and methods

This study is an investigator’s initiated, perspective, local, single-institution, single-arm, open-label, phase I/II trial testing the effect of Durvalumab and weekly paclitaxel for the management of metastatic TNBC. The study followed good clinical practice (GCP) and was conducted according to the declaration of Helsinki.

### Patient recruitment and consenting

This study recruited female patients with histologically confirmed metastatic TNBC, whether de novo or recurrent disease. It included only patients accepting to sign a clear and explicit informed written consent to be enrolled in this trial, who are 18–65 years old, with Eastern Cooperative Oncology Group (ECOG) performance status of 0–1, measurable disease per Response Evaluation Criteria in solid tumors (RECIST) version 1.1, a life expectancy ≥ 12 weeks, normal organ and marrow functions, and with non-reproductive potential or willing to use strict contraceptive methods. One line of chemotherapy in the metastatic setting was allowed with no prior targeted therapy. On the other hand, patients were excluded if they were on immunosuppressive or previous immune checkpoints-targeting therapies, had a history of immune-related diseases, or symptomatic/uncontrolled brain metastasis.

### Study design

The study was designed to be in two phases, a dose de-escalation phase and an expansion phase. In the dose de-escalation phase, the tolerability of the standard dose of 80 mg/m^2^ of paclitaxel in combination with Durvalumab was planned to be tested in three patients, and in case it was not tolerable, then paclitaxel would be decreased to 70 mg/m^2^ on three patients. Similarly, in case of significant toxicity with 70 mg/m^2^ paclitaxel, the dose would be decreased to 60 mg/m^2^ on three patients. The expansion phase was planned after (Supplementary Fig. 1).

The primary objective of the study was to test the safety of the Durvalumab and Paclitaxel combination, while the secondary objectives were to evaluate the objective response rate (ORR) and to assess the progression-free survival (PFS) and overall survival (OS). In addition, we correlated baseline PD-L1 expression as well as TIL infiltration with the response.

The treatment regimen was composed of a single cycle of paclitaxel alone for immunological priming and to exclude patients who cannot tolerate paclitaxel alone. This is followed by five cycles of a combination of weekly paclitaxel 80 mg/m^2^ given intravenously (IV) on days 1, 8, and 15 and Durvalumab (750 mg as fixed-dose) given IV with Paclitaxel on days 1 and 15 of every 28 days' cycle. Upon the completion of the Paclitaxel Durvalumab cycles, Durvalumab was given alone every 14 days with the same dose until disease progression or unacceptable toxicity (Supplementary Fig. 2).

In order to overcome the potential pseudoprogression, certain patients were allowed to continue treatment beyond disease progression, as assessed by RECIST 1.1. However, treatment was stopped when disease progression is confirmed on a subsequent CT scan.

### Clinical and radiological assessment and follow-up

Toxicity was assessed using Common Terminology Criteria and Adverse Events (CTCAE) version 5.0. Patients were followed up by clinical examination, and a CT scan was performed every 8–10 weeks to monitor for tumor response and/or disease progression. The ORR and duration of response were assessed per the Response Evaluation Criteria in Solid Tumors, version 1.1^23^. Calculation of total tumor burden was made by volumes summation of all measurable targets using the equation:

$$Volume=6\uppi \times \mathrm{LD}\times \mathrm{SD},$$

where LD is the longest diameter and SD is the shortest diameter.

### Histology and immunohistochemistry

PD-L1 was assessed by immunohistochemistry using four microns' thickness sections from formalin-fixed paraffin-embedded (FFPE) tissue blocks of core biopsy taken from the patients enrolled in this trial. Immunostaining was done using a fully automated Ventana Benchmark Ultra system and Ventana compatible reagents, including the anti-PD-L1 antibody (SP263 clone).

A pathologist (AT) scored PD-L1 on tumor cells in 5–10% increments. PD-L1 expression in tumor cells is equivalent to the total proportional score (TPS) typically used with anti-PD-L1 antibody clone SP263 to correlate with response to Durvalumab in lung and bladder cancer. Tumor-infiltrating lymphocytes (TIL) were scored as low, medium, or high based on the Salgado et al. method^24^. Briefly, TIL occupying less than 10% of the examined field was considered low, while an infiltration occupying 10–40% of the field was considered moderate, and above 40% was considered high.

### Statistical analysis

Cox proportional hazards regression model was done using JMP^®^ from SAS Institute (Cary, USA). Kaplan–Meier curves were made, and the median survival was calculated using GraphPad Prism 5 or JMP^®^. A p-value of < 0.05 was considered significant.

### Ethics approval and consent to participate

﻿The study was approved by the Institutional Review Board (IRB) of King Faisal Specialist Hospital and Research Centre, mainly composed of the Research Ethics Committee (REC) and the Clinical Research Committee (CRC), who approved the trial, the consent form and the treatment protocol (RAC# 2151-196). The study and the treatment protocol were further approved by the collaborating company, AstraZeneca (ESR 14-10649) and the national health authority, Saudi Food and Drug Administration (SFDA), under Clinical Trial Registry (#160119020). The study was conducted according to these guidelines and regulations.﻿The trial was registered in the US NIH clinical trial registry (clinicaltrial.gov): NCT02628132 on 11/12/2015

## Results

### Enrollment and patients characteristics

Between Feb 2017 and July 2019, 24 patients were recruited, among which only 21 patients were committed to the treatment while three withdraw from the trial to seek treatment abroad (n = 2) or because they found the weekly treatment protocol inconvenient (n = 1). In the initial cycle of paclitaxel and before any dose of Durvalumab, five more patients were removed from the study due to tumor progression (n = 3) or intolerance to paclitaxel alone (n = 2). Two patients were further removed from the analysis as they progressed (locally and radiologically confirmed) and thus removed from the study a few days after receiving one dose only of Durvalumab. Patients who completed at least one cycle of the combination therapy (14 patients) were included in the final analysis (Supplementary Fig. 3). Recruitment was closed as of July 2019; thus, results in this report are final for this trial.

Most of the patients were relatively young (median age was 42 years), and 43% of patients were younger than 40 years old (Table 1). This relatively young age of patients is consistent with known characteristics of TNBC^25^ and the median age of breast cancer patients in Saudi Arabia and the Middle East in general^26^. In agreement, the vast majority (81%) of patients were premenopausal, and most of them had ECOG 0. Three patients (21%) had TNBC with metaplastic histology. The majority of patients (86%) received the combination of Durvalumab and Paclitaxel as a first-line in the metastatic setting. Of note, most of the cases (71%) were recurrent rather than de novo metastatic TNBC (Table 1), which implies that they have received chemotherapy previously in the neoadjuvant or adjuvant setting while being treated for the local disease.

**Table 1:** Patients characteristics.

	(%)
**Age (**years**)**	
< 42	8 (57)^a^
≥ 42	6 (43)
**Menopause**	
Pre	10 (71)
Post	4 (29)
**Histology**	
Not metaplastic	11 (79)
Metaplastic	3 (21)
**ECOG**	
0	11 (79)
1	3 (21)
**Prior line of therapyb**	
0	12 (86)
1	2 (14)
**Presentation**	
De novo	4 (29)
Recurrent	10 (71)

^a^Percentage of cases.

^b^In the metastatic setting.

### Safety

In the dose de-escalation phase of the study, the combination of Durvalumab and Paclitaxel at 80 mg/m^2^ was found tolerable with no dose-limiting toxicity. Therefore, the trial moved to the expansion phase using paclitaxel at a dose of 80 mg/m^2^. Similarly, in the expansion phase, the combination of Durvalumab and Paclitaxel was found to be safe and tolerable. Treatment-emergent adverse events (AEs) were observed in 10 (71%) patients, regardless of grade, while grade 3–4 AEs were observed in 3 (21%) patients (Table 2). All AEs, regardless of grade, eventually resolved by temporarily holding treatment and/or the use of appropriate AEs management. There was one case of Durvalumab discontinuation due to AE (skin reactions, see below). There was neither death due to therapy nor AEs that led to dose reduction.

**Table 2:** Patients (%) reporting adverse events (AEs).

Type of AEs	No. (% of total n = 14)
Any AEs	1 (7)
Serious AE (grade ≥ 3)	3 (21)
AEs leading to discontinuation	1 (7)
AEs leading to dose reduction	0 (0)
Death due to combination therapy	0 (0)
Death due to disease progression	9 (64)

The most common (incidence > 1%) AEs, regardless of grade, were headache and peripheral sensory neuropathy, which happened in 4 (29%) patients (Table 3). The second most common AEs were fatigue (21%) and skin rash (21%). Other AEs were alopecia, anemia, neutropenia, nausea, diarrhea, pruritus, palpitation, and weight gain. The most common grade 3/4 treatment-related AEs were headache and anemia, which each happened in 2 (14%) patients. Less common grade 3–4 AEs were neutropenia, fatigue, and skin rash. All the observed AEs were previously known to be related to Durvalumab or Paclitaxel or both, and no new safety signals were detected using the combination.

**Table 3:** Adverse events experienced by any of the patients in this trial.

Adverse event (AE)	All grades AEs # (%)^a^	Grades ≥ 3 AEs # (%)	Known AEs?^b^	
			P	D
Neuropathy	4 (29)	0 (0)	**√**	√
Headache	4 (29)	2 (14)	**√**	**√**
Anemia	2 (14)	2 (14)	**√**	**√**
White blood cells decreased	1 (7)	1 (7)	**√**	√
Neutropenia	1 (7)	1 (7)	**√**	
Eosinophilia	1 (7)	0 (0)	√	√
Fatigue	3 (21)	1 (7)	**√**	**√**
Fever	1 (7)	0 (0)	**√**	**√**
Sore throat	1 (7)	0 (0)	√	√
Edema	1 (7)	0 (0)	√	**√**
Diarrhea	2 (14)	0 (0)	**√**	**√**
Constipation	1 (7)	0 (0)	**√**	**√**
Colitis	1 (7)	0 (0)	√	√
Increased ALT/AST enzymes	1 (7)	0 (0)		**√**
Dyspnea	1 (7)	0 (0)		**√**
Cough	1 (7)	0 (0)		**√**
Skin rash	3 (21)	1 (7)	**√**	**√**
Pruritus	2 (14)	0 (0)		**√**
Vaginal dryness	1 (7)	0 (0)	√	
Alopecia	2 (14)	0 (0)	**√**	√
Nail infection	1 (7)	0 (0)		√
Nail changes	1 (7)	0 (0)	√	
Palpitation	2 (14)	0 (0)		√
Wt. gain	2 (14)	0 (0)		√
Hoarseness of voice	1 (7)	0 (0)	√	√
Hypophysitis	1 (7)	0 (0)		√
High cholesterol	1 (7)	0 (0)		√
Myalgia	1 (7)	0 (0)	**√**	√
Arthralgia	1 (7)	0 (0)	**√**	**√**
Back pain	1 (7)	0 (0)		**√**
Dry eye	1 (7)	0 (0)	√	

^a^% from total of 14 patients. Of note, the two patients who received one dose of Durvalumab only did not report any AEs

^b^Known AE from drug brochure for either Paclitaxel (P) or Durvalumab (D) or both. Bold AEs are well-known or common (> 10% of patients had experienced it in previous monotherapy trials).

Adverse events of special interest (AESIs) of any grade occurred in 2 (14%) patients. One patient presented with nocturnal seizures, forceful tonic posturing, unrolling of eyes, and urinary incontinence. MRI of the brain revealed subcortical FLAIR hyperintensity in the bilateral occipital lobe that is highly suggestive of posterior reversible encephalopathy syndrome (PRES). The neurophysiology report (using EEG) showed an abnormal study with evidence of left temporal cerebral dysfunction. The patient was started on Levetiracetam (Keppra^®^) 500 mg twice daily, and Durvalumab was put on hold for 4 weeks. Later on, treatment with Durvalumab resumed, and the patient is still doing well with no further neurological AEs. The second patient presented with painful, itchy multiple dusky red and violaceous papules described as lichenoid drug eruptions (LDE) that responded to steroids and cyclosporine. Durvalumab was discontinued permanently.

The above AESI happened in patients after 2 years of treatment, i.e., in the two patients who did very well (no relapse) and thus kept receiving Durvalumab for more than 2 years. On the other hand, patients who received only one dose of Durvalumab did not report any AEs.

### Clinical activity

**Figure 1:**
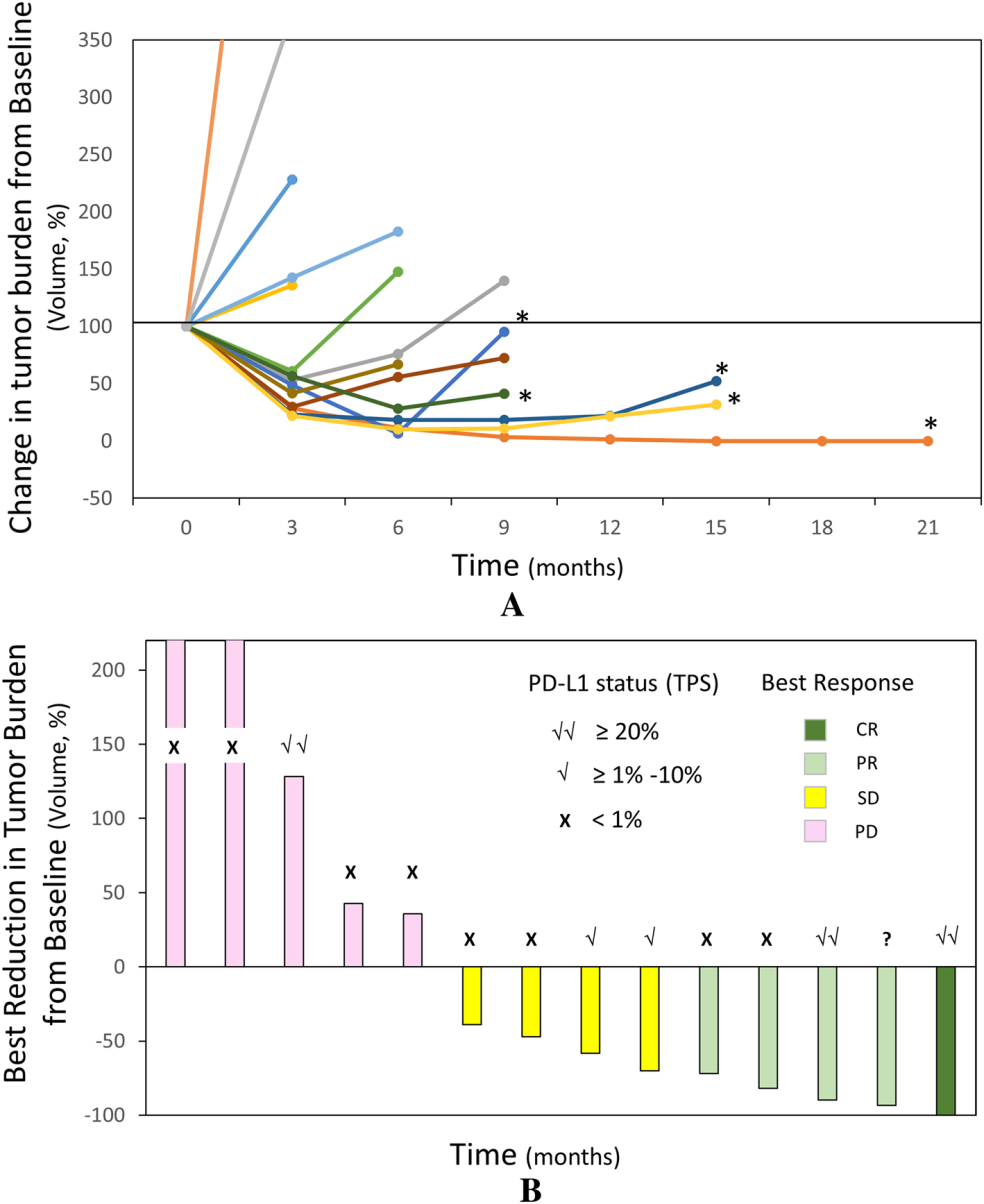
Change of tumor burden in patients after starting treatment. Timeline for change in tumor burden (total volume) based on tumor dimensions provided in patients' CT scans. *Patients with confirmed partial or complete response. Tumor burden is followed until discontinuation of therapy (n = 13) or complete response (n = 1).

With a median follow-up of 24 months, the objective response rate (ORR) was 36% (confirmed), and the response was durable, with a median of 10 months and a disease control rate (DCR) of 64% (Fig. 1 and Table 4). Responders included one patient with complete response (CR), which was described previously^27^ and four patients with partial response (PR) (Table 4).

**Table 4:** Response to Durvalumab and Paclitaxel combination.

Response	No. of pts	(% of total n = 14)^a^	(% of total n = 19)^d^
**Best overall response**^**a**^			
CR	1	(7)	(5)
PR	4	(29)	(21)
SD	4	(29)	(21)
PD	5	(36)	(53)
Overall response rate (ORR)^b^, confirmed	5	(36)^c^	(26)
Unconfirmed ORR	8	(57)	(42)
Disease control rate (DCR)	9	(64)	(47)

^a^Determined as per RECIST 1.1.

^b^ORR = CR + PR.

^c^Confirmed ORR refers here to two consecutive PR or CR determined by CT with a minimum of 1 month apart while a PR followed by SD is considered SD.

^d^With the addition of five patients who relapsed while on the paclitaxel alone cycle.

To avoid the bias due to the five patients who were removed due to progression while in cycle 1 of paclitaxel alone (n = 3) or days after receiving Durvalumab (n = 2), we included these patients (n = 5) to the 14 patients receiving the combination as intent to treat (ITT) group (n = 19). In this ITT group, the confirmed ORR was 25% (Supplementary Fig. 4).

**Figure 2:**
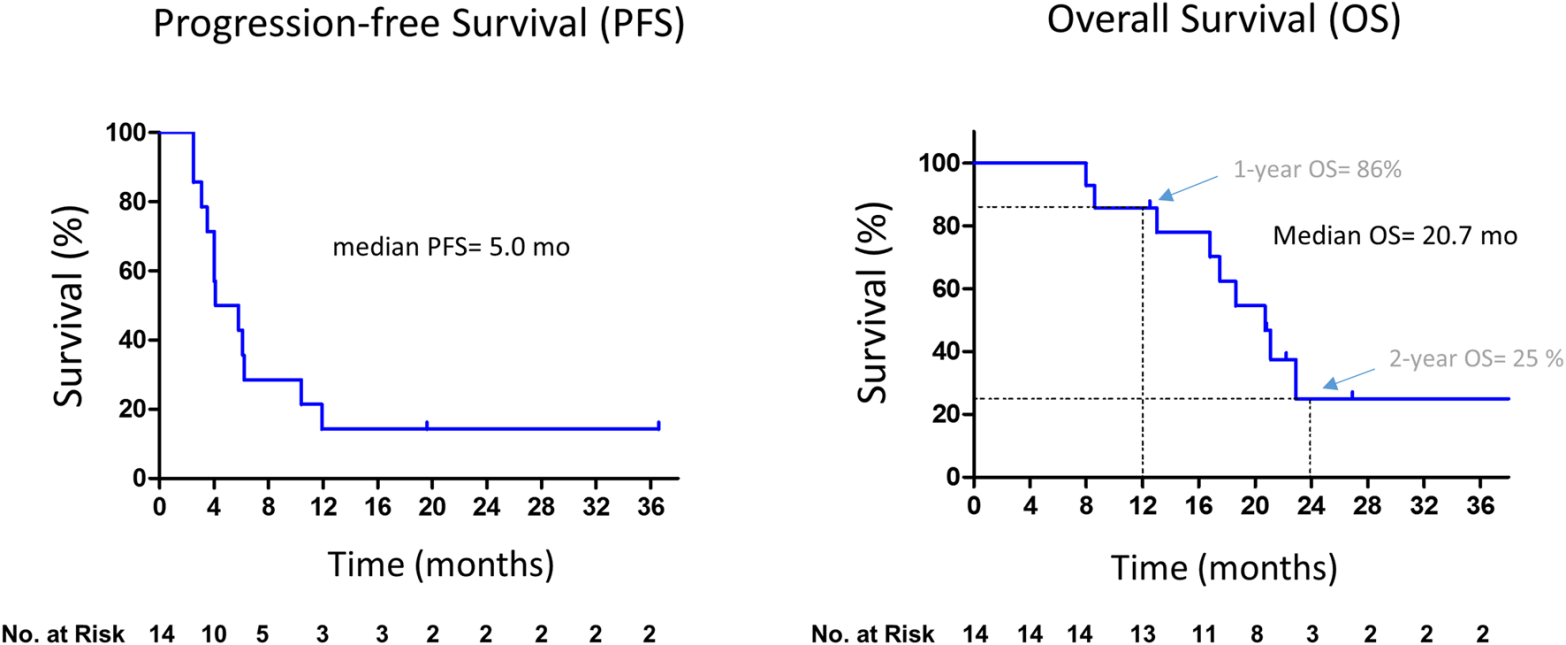
Progression-free and overall survival of patients. Kaplan–Meier survival curves showing progression-free survival (PFS) and overall survival (OS) of patients.

The median progression-free survival (PFS) and overall survival (OS) were 5.0 and 20.7 months, respectively (Fig. 2). While in the ITT population, the median PFS and OS were 4 and 20.7 months, respectively.

### Biomarkers analysis

In a search for tumor biomarkers, we examined the tumoral PD-L1 expression and density of stromal TIL. PD-L1 was expressed (> 1% of tumor cells) in 5 (36%) of patients. There was a trend towards a correlation between PD-L1 expression and longer PFS, although it did not reach statistical significance per univariate Cox survival regression analysis (p = 0.111). Similarly, the segregation of the Kaplan–Meier survival curves based on tumoral PD-L1 expression status was not significant (Supplementary Fig. 5). In general, there was low TIL in 50% of patients, and there was no significant correlation between TIL and PFS (Supplementary Table 1).

## Discussion

Metastatic TNBC is one of the worst types of breast cancer and the lowest survival^28^. A taxane like paclitaxel is among the best agents to manage metastatic TNBC; however, survival is still short^15^. In this investigator-initiated, single-arm trial, we have tested the safety and efficacy of a combination of weekly Paclitaxel with Durvalumab. The current study, for the first time, show that Paclitaxel and Durvalumab combination is safe with minimal manageable AEs, rare dose-limiting toxicity, and no therapy-related mortality, allowing possibly for additional therapeutic agents to this combination.

Incidence of all AEs, including grade 3–4 toxicities, was generally similar or even lower than previously reported for paclitaxel alone, with the exception of peripheral sensory neuropathy and headache. For example, diarrhea and alopecia were experienced by 14% of patients in this trial as compared to 11–27% reported for diarrhea, and 13–50% previously reported for alopecia as an AEs from Paclitaxel alone^29–31^. In agreement, AEs in this trial were generally lower than previously reported AEs with combinations involving chemotherapy with a checkpoint inhibitor. For example, in the impassion 130, alopecia and diarrhea were reported in 56% and 32%, respectively, of patients taking the combination of Nab-paclitaxel and the anti-PD-L1, atezolizumab^10^. Similarly, in Impassion 131^32^, alopecia and diarrhea were reported in 59% and 30%, respectively, of patients taking a combination of atezolizumab and paclitaxel compared to 14% for both AEs in this trial. The lower dose of paclitaxel used (80 mg/m^2^) compared to paclitaxel 90 mg/m^2^ used in impassion 131 and 100 mg/cm^2^ of nab-paclitaxel used in impassion 130 could possibly contribute to this lower incidence of AEs in this trial. In addition, patients in this trial were younger (median age was 42 vs 55 years in impassion 130 and impassion 131^10,32^), possibly contributing to lower incidence of AEs like diarrhea^33^.

On the other hand, peripheral neuropathy and headache were experienced by 29% of patients in this trial, which is relatively higher than the previously reported incidence rate of 14–19% for neuropathy and 7–18% for headache for paclitaxel alone^29–31^. Of note, predisposing diseases like diabetes mellitus, which is significantly more common in Saudi Arabia^34^, may contribute to a higher incidence of peripheral neuropathy. In addition, while headache is one of the reported AEs for both paclitaxel and Durvalumab, it is also likely to be due to the antiemetic Granisetron (2 mg), which is routinely given paclitaxel to alleviate chemotherapy-induced nausea, or the combination of these agents.

We carefully monitored patients for pneumonitis, colitis, thyroiditis, and immune-related hepatitis as possibly life-threatening AEs that could be induced by both agents. While pneumonitis was not observed in any patient, colitis was experienced by one patient (grade 2), and it resolved later on by temporarily holding the trial therapy. Surprisingly, we did not observe any immune-related hepatitis, hypothyroidism, or hyperthyroidism in any patient despite routine check of thyroid and liver function tests. Of note, four patients had hypothyroidism before enrollment in this trial. In comparison, 20% of patients developed either hypothyroidism (14%) or hyperthyroidism (6%) in Impassion 131^32^. Similarly, 20% of patients developed either hypothyroidism (15%) or hyperthyroidism (5%) from a combination of pembrolizumab and chemotherapy in KEYNOTE-355^35^. On the other hand, during this trial two patients gained weight, and one patient complained of hoarseness of voice.

The observed lichenoid drug eruptions (LDE) in one of the patients in this trial is previously well-known as a rare AE for PD-1/PD-L1 therapies^36^, which also seems to be associated with taxane combinations^36–38^. Similarly, the encephalopathy, specifically the PRES experienced by the other patient, is another rare AE for PD-1/PD-L1 therapies^39^ and checkpoint inhibitors in general^40^. Duration of treatment seems to be important as both AEs (LDE and PRES) happened in patients who have been taking the combination for more than 2 years, while patients who received only one dose of Durvalumab did not report AEs.

Due to the trial design based on an initial cycle of paclitaxel alone, some patients had to be excluded due to disease progression, which allowed for a degree of bias. Therefore, we calculated ORR with and without the relapsing patients to minimize this effect, which turned 25 and 36%, respectively. The confirmed ORR of 25–36% seen with Durvalumab and Paclitaxel combination in this trial was noticeably above the ORR of 5–21% previously reported with anti-PD-L1 monotherapies^11,13^ and possibly close to the 22–32% ORR previously reported in paclitaxel alone arm of several trials^29–31,41^. However, the ORR in this trial was below the ORR of 39–56% that has been observed with the combination of the anti-PD-L1, Atezolizumab, and Nab-paclitaxel^10,42^. Notably, one patient had a complete response, her case was previously described^27^, and one patient is still with stable disease and doing fine. Of note, we had to use the confirmed ORR in this trial, a typical situation in phase I studies as per RECIST v1.1 recommendations^23^.

In this trial, the combination of Durvalumab and Paclitaxel had a median PFS of 4.0–5.0 months, which is not significantly different from historical data on median PFS using paclitaxel alone (3.5–5.3 months)^29–31,41^. On the other hand, the overall survival (OS) with the Durvalumab/paclitaxel combination of 20.7 months was higher than the previously reported median OS with Paclitaxel alone (i.e., 12.6–16.3 months)^29–31,41^ or the median OS of 13.1 months for metastatic TNBC in general regardless of treatment regimen^43^. However, this increase in OS might not be meaningful due to the small number of patients and lack of a control arm. In general, despite small sample size restricting the ability to draw meaningful conclusions, efficacy data in this trial were close to the recently reported results for Impassion 131^32^, which reported a median PFS of 5.6 vs. 5.7 months and OS of 22.8 vs. 19.2 months for paclitaxel + placebo compared to paclitaxel + atezolizumab in ITT population.

This study admittedly has limitations, including the small sample size that did not reach the initially planned. In addition, the population of patients was local from one geographical area. However, despite limitations, the good safety profile for the paclitaxel and Durvalumab combination is encouraging and suggests that there is a room for additional agents that can be added to the Paclitaxel/Durvalumab combination as a feasible way to improve the outcome of metastatic TNBC in the future.

In conclusion, this is the first study to report on the Durvalumab and Paclitaxel combination in metastatic TNBC. The study shows that the combination was safe. Further large prospective randomized trials can be conducted in the future to more accurately determine the efficacy of this combination.

## Supplementary Information

Supplementary Figures.

Supplementary Table 1.

## Data Availability

The datasets generated during and/or analyzed during the current study are included in this published article (and its supplementary information files), otherwise available from the corresponding author on a reasonable request.

## Abbreviations

PD-L1: Programmed death ligand-1
TNBC: Triple-negative breast cancer
ORR: Objective response rate
PFS: Progression-free survival
OS: Overall survival
