# Supplementary material for: Weekly Paclitaxel given concurrently with Durvalumab has a favorable safety profile in triple-negative metastatic breast cancer
Source: Sci Rep. 2021 Sep 27;11:19154. doi: 10.1038/s41598-021-98113-6 (PMC8476586; doi:10.1038/s41598-021-98113-6)
Supplement: Supplementary file 1 — Supplementary Figures. [file 41598_2021_98113_MOESM1_ESM.pdf]

Weekly Paclitaxel given concurrently with Durvalumab has a favorable safety profile in triple-negative metastatic breast cancer

Hazem Ghebeh BPharm, PhD[1,2]*, Adher Al-Sayed MD[3], Riham Eiada MD[4], Leilani Cabangon BN[3], Dahish Ajarim MD[3], Kauser Suleman MD[3], Asma Tulbah MD[5], and Taher Al-Tweigeri MD[3]*

Affiliations :1Research Centre, [2]College of Medicine, Al-Faisal University, Riyadh, Saudi Arabia, [3]Oncology Centre, [4]Department of Radiology, [5]Department of Pathology and Laboratory Medicine, King Faisal Specialist Hospital and Research Centre, Riyadh, Saudi Arabia.

* CORRESPONDENCE:

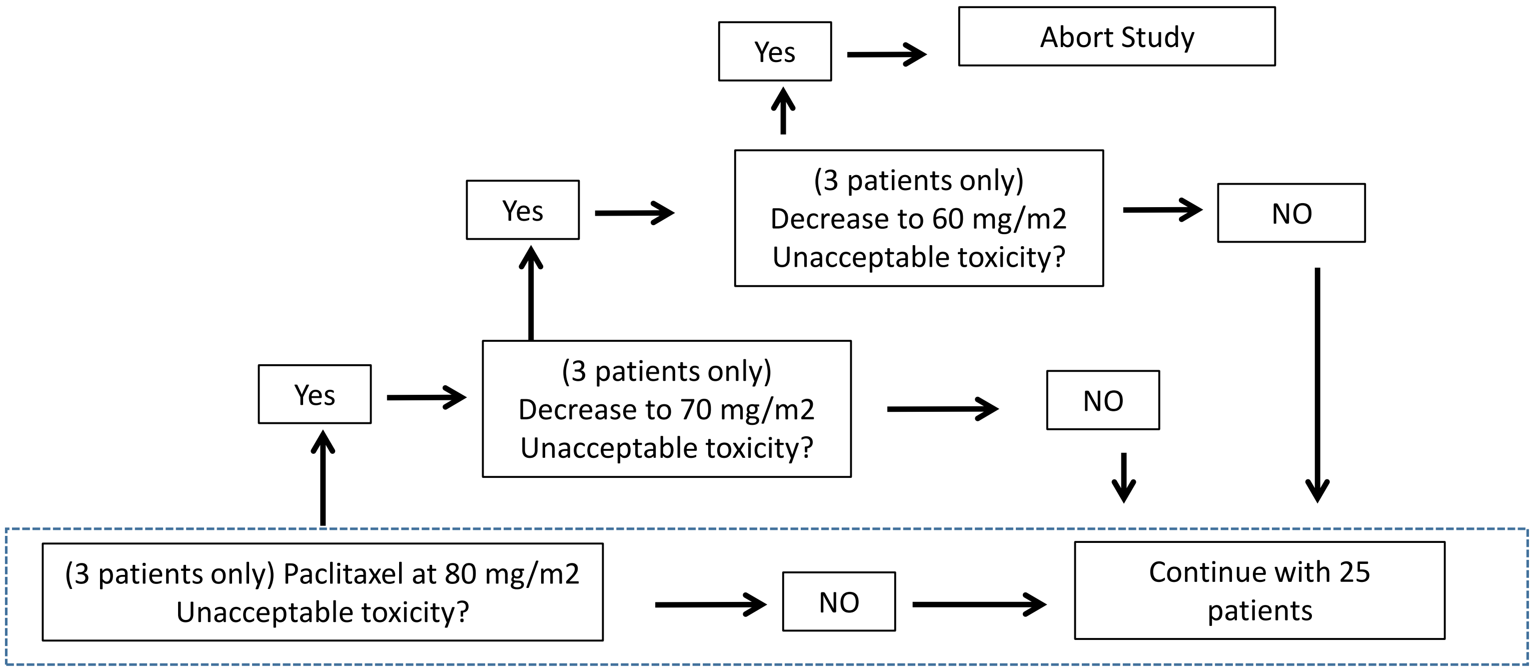

Supplementary Figure 1: Study Design. The study was designed into the dose de-escalation phase and expansion phase.

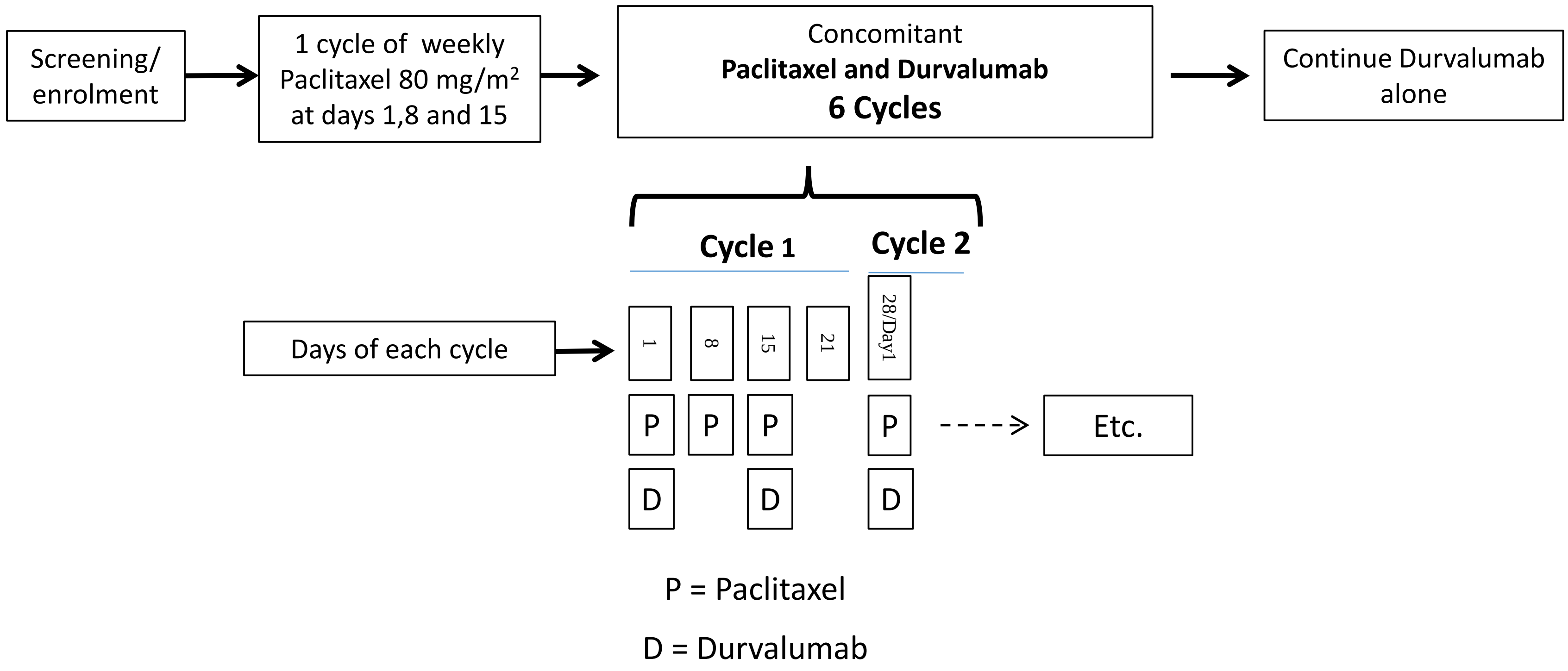

Supplementary Figure 2: The treatment regimen. The treatment regimen starting with 1 cycle of paclitaxel alone followed by 5 cycles of concurrent Paclitaxel and Durvalumab. Upon completion of combination cycles, Durvalumab will be given until disease progression or limiting toxicity.

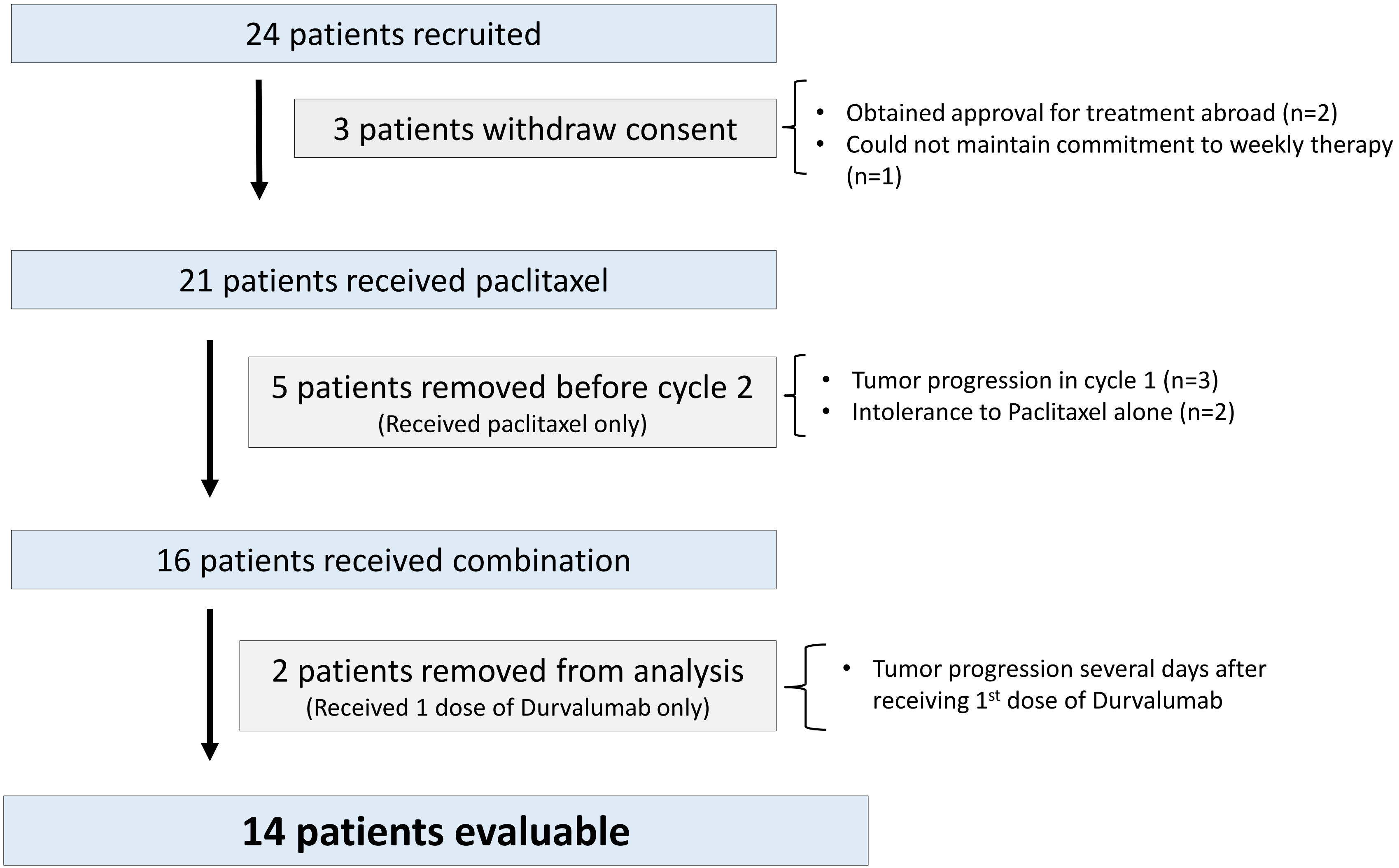

Supplementary Figure 3: Trial recruitment diagram. Schematic diagram showing the recruitment status.

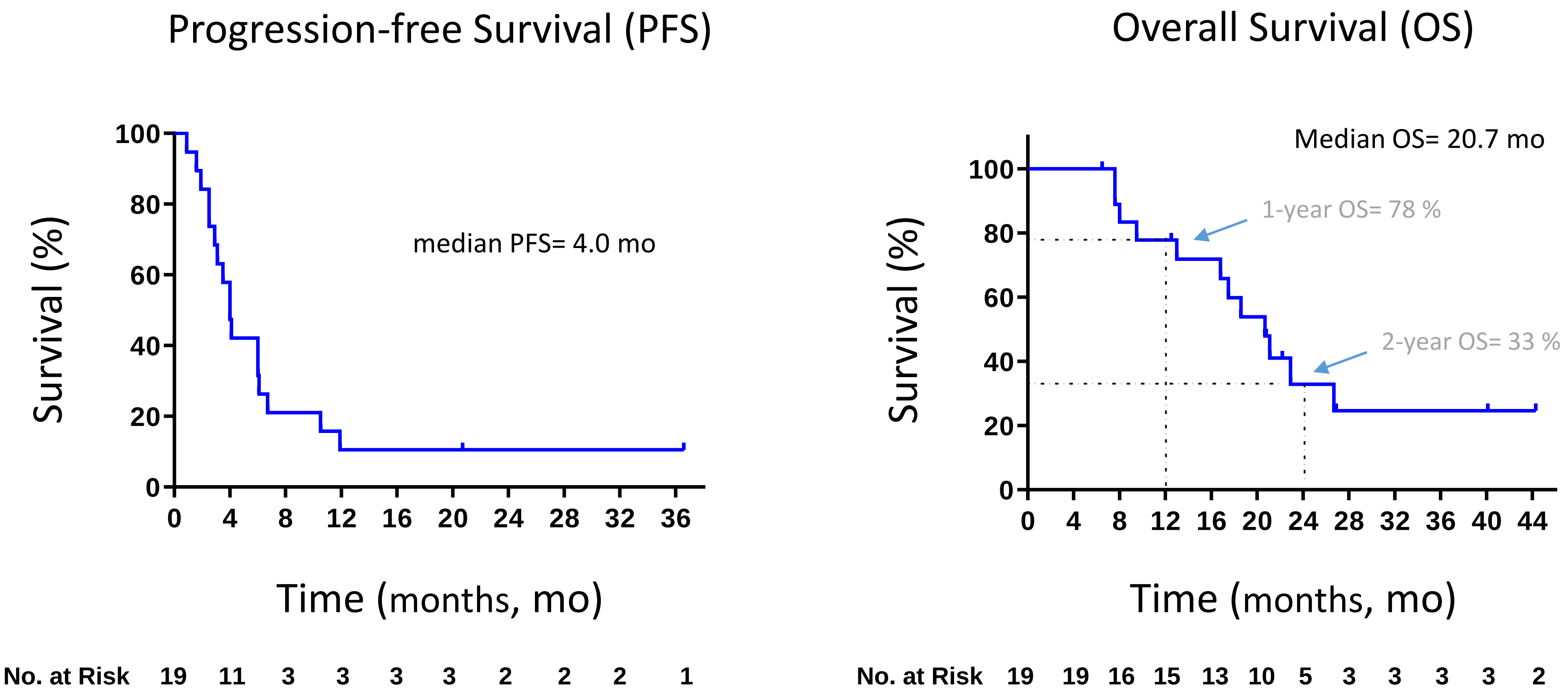

Supplementary Figure 4: Progression-free and overall survival. Kaplan–Meier survival curves showing progression-free survival (PFS) and overall survival (OS) of the total number of patients (n=19), including those who received paclitaxel alone, due to progression during the first cycle (n=5) in addition to patients treated with the combination of paclitaxel and Durvalumab (n=14).

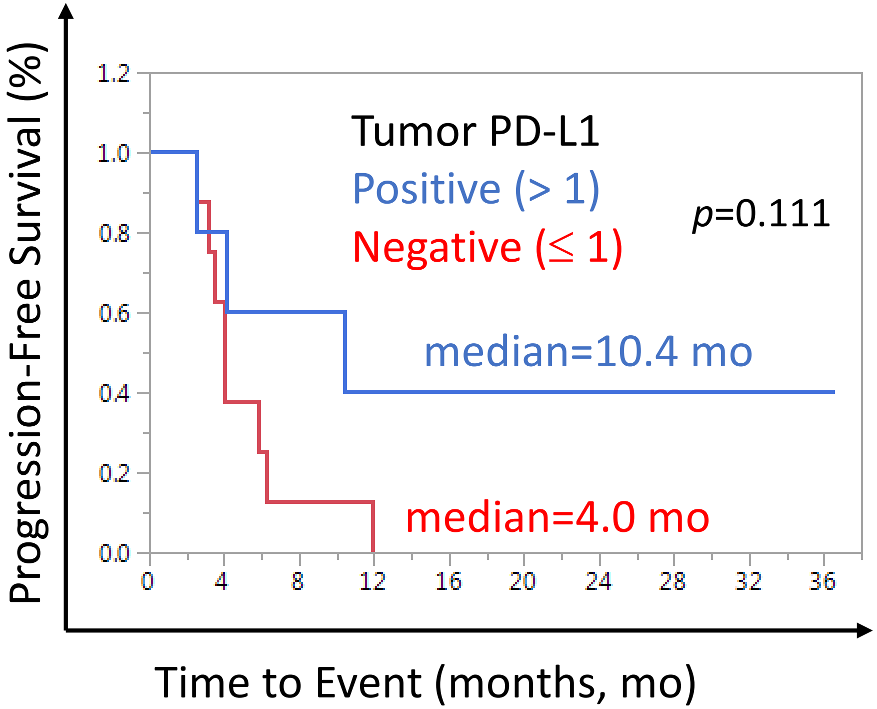

Supplementary Figure 5: Progression-Free Survival (PFS). Kaplan-Meier survival estimate based PD-L1 status. * indicate significance as tested by log-rank test.
