# Supplementary material for: Weekly Paclitaxel given concurrently with Durvalumab has a favorable safety profile in triple-negative metastatic breast cancer
Source: Sci Rep. 2021 Sep 27;11:19154. doi: 10.1038/s41598-021-98113-6 (PMC8476586; doi:10.1038/s41598-021-98113-6)
Supplement: Supplementary file 2 — Supplementary Table 1. [file 41598_2021_98113_MOESM2_ESM.docx]

**Supplementary Table 1. Univariate Cox proportional hazard regression analysis** of PD-L1 and lymphocytic infiltration with Progression-free survival (DFS) of the trial patients (n=14).

|  | **Relapse** | |  | **Univariate** |  |
| --- | --- | --- | --- | --- | --- |
|  | **-** | **+** |  | ****P*** |  |
| **PD-L1 Tumor** |  |  |  |  |  |
| > 1% | 0 (0)^♣^ | 8 (100) |  |  |  |
| ≤ 1% | 2 (40) | 3 (60) |  | 0.112 |  |
|  |  |  |  |  |  |
| **Lymphocytic Infiltration** |  |  |  |  |  |
| High | 1 (14) | 6 (86) |  |  |  |
| Low | 1 (17) | 5 (83) |  | 0.672 |  |
|  |  |  |  |  |  |

**Abbreviations**: (+ and -) are number of positive and negative patients, ^♣^Numbers between brackets are the percentages of patients, **P* values in bold represent significant data.
